# Spotlight on early-career researchers: an interview with Marie Heffern

**DOI:** 10.1038/s42003-018-0057-z

**Published:** 2018-06-25

**Authors:** Dominique Morneau

**Affiliations:** Communications Biology, https://www.nature.com/commsbio

## Abstract

Marie Heffern began her independent career at UC Davis in July 2017. In this short Q&A (the first of a new series highlighting early-career researchers), she tells us about her experience as a young researcher and the advice she has for her younger self.

**Figure:**
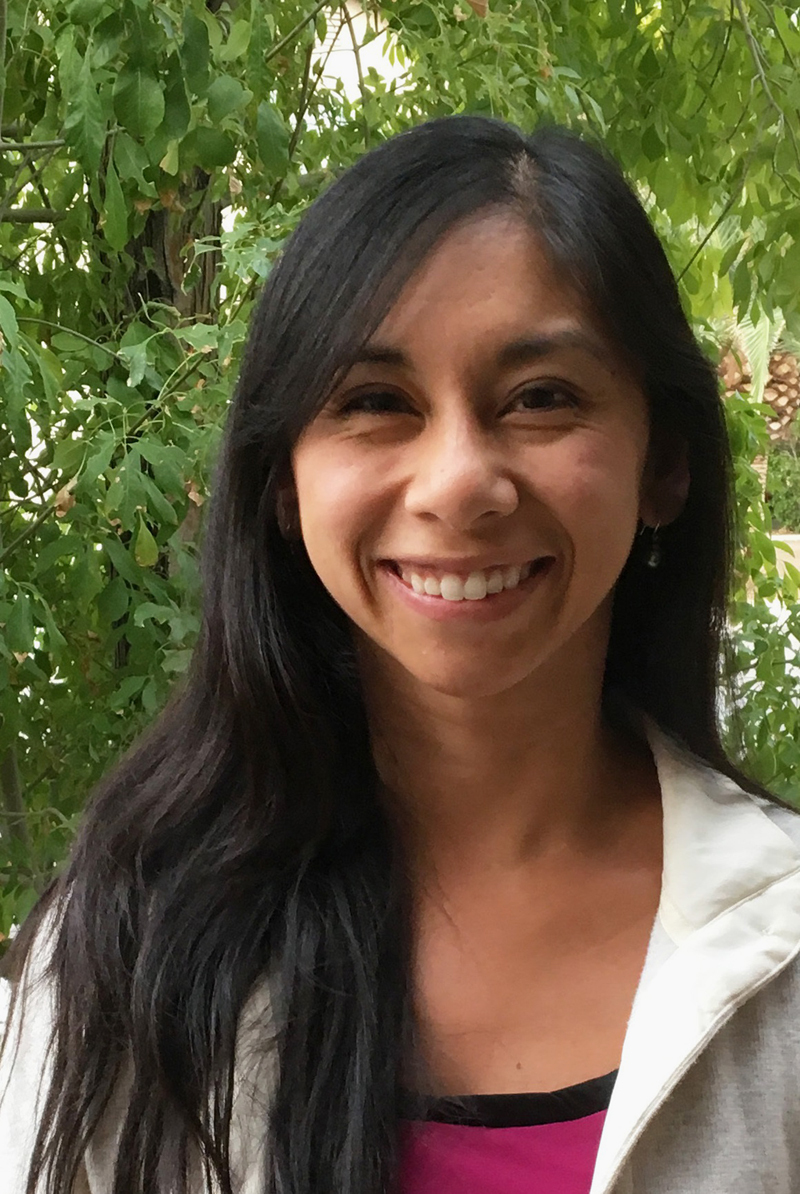
Marie Heffern

**Please tell us about your research interests.**

My broad research interest is to investigate metals in medicine at multiple scales, that is, looking at bioinorganic connections from the molecular level to the whole-organism. I am interested in the moving parts and dynamics of metals in complex biological environments. As a starting point, we are trying to understand the role of metals in hormone biology as it applies to nutritional and metabolic disorders including obesity, liver disease, and diabetes.

**What has your journey been to this point?**

My journey has been a steady development toward letting my passions drive me to push my limits rather than magnifying the barriers that fears and insecurities can put in the way of my pursuits. When I think through my personal history, I recall many instances when I have felt like I was starting a race at the back of the pack. The key influential moments and people in my life are those who have inspired me to focus forward on the next step rather than worry that I am starting behind from everyone else.

Reminiscing on how I started in my past research labs highlights this concept. When I was an undergraduate, I felt like I was starting research late in college as compared to my chemistry peers. Nonetheless, my undergraduate research advisor, Prof. Richard Brutchey gave me trust and independence that motivated me to take ownership of my projects. When I joined Prof. Tom Meade’s lab for graduate school, I was eager to work on projects at the interface of chemistry and biology despite having only taken one biology course (“Cell Biology and Physiology”) in college. He was not fazed by the fact that I did not know the difference between a western blot and a DNA gel or the structures or names of amino acids. He supported my ambition and applauded my desire to get out of my comfort zone for the sake of pursuing the scientific discoveries that fascinated me. In my postdoc, I had expressed my interest to Prof. Chris Chang in pushing my scientific limits toward researching bioinorganic chemistry in whole animals. While I had never worked with research animals at the time, he did not hesitate to support me in the projects and collaborations that could immerse me in a productive learning environment. These are but a few examples in a broader experience that motivate me to not let where I start diminish my confidence and drive toward what I strive to accomplish in the future.

I would not say that my journey to this point makes me fearless, but rather, it has empowered me to choose the level of influence that fear and worry (which sometimes feel inevitable) have on my self-assessment and decisions as compared to the influence of my own passions, enthusiasm, and curiosity. I started my lab at UC Davis less than a year ago, and it has been exciting to not only apply these lessons to my own career but to also figure out how I can instill these values and confidence in those I mentor.

**What are your predictions for your field in the near future?**

Recent years have seen a new advent in genomics, bioinformatics, and chemical biology tools for high-throughput evaluations of complex systems. I think these developments will have a large impact on how we study metals in medicine in health. While much of previous work has focused on expanding serendipitous systems like cisplatin or understanding the interactions of metals with purified and isolated systems, these advances will open up opportunities to study complex environments, as well as discover new metal-binding and metalloregulatory biomolecules that are difficult to predict with traditional methods. These advances will allow for connections to be drawn from observed phenomena in areas such as nutrition to molecular pictures.

**Can you speak of any challenges that you have overcome?**

On a more general note, as I alluded to in the previous question, I have struggled with confidence rooted in a feeling like I was starting a step behind my peers. Regarding my journey toward an independent career, I had felt insecure in my ability to come up with original proposals and ideas. One of the most memorable points in my career toward overcoming this was when I had asked one of my scientific heroes, Prof. Kathy Franz, how she got started and how she comes up with original ideas, and she said, “I don’t know how one ‘comes up’ with ideas. I just like to pursue questions that I have.” Her response provided a different perspective that highlighted the personal nature of independent research—that excellent research does not have to come from saying “let me think of a brand new idea that no one else has thought of.” Rather beautiful discoveries can come from curious minds trying to understand and uncover the vast unknowns of our universe, and a researcher’s questions are colored by their own unique background.

More specific to being an early-career researcher, a major challenge has been time management. This has less to do with the quantity of items on my to-do list, but rather, not being able to properly assess how long some of those items take. Leading up to my postdoc, I grew increasingly effective in my time management as I gained more accuracy in estimating how long certain procedures in the lab would take. However, when I started my position, there were a LOT of new categories of tasks. Teaching for the first time, I had no idea how long lecture preparations or writing exams and homework assignments could take. Meetings and negotiations with vendors were more time-consuming than I had anticipated. I had to learn the balance between penny-pinching and understanding that time is also money. While I am not sure it is fair to say I have overcome this challenge, I have learned the importance of seeking the support and advice of my colleagues, making deliberate efforts to protect my own time, and realizing that “buffer time” in my schedule is a necessity.

**What advice would you give to your younger self?**

First, focus on your passions and unique curiosities. Do not worry about fitting into a box or trying to be someone you are not. You will operate more effectively when you are doing what you love.

Second, surround yourself with people who you respect, because you can easily become like those you surround yourself with.

Third, do not underestimate the value of a strong network. Beyond my direct advisors and labmates, I have been surprised with the support and influence that I have experienced from more distant connections and advocates.

**(Bonus question). What is your favorite metal?**

Cobalt. I am fascinated by its biologically flexible oxidation states and ligand affinities. Its behavior is highly sensitive to its surrounding environment and a good example of why we must look beyond identifying the metal in a biomolecule but also understand its context and dynamics. Additionally, its name has a fun root as a metal of mischief. “Cobalt” comes from “Kobold”, the name of a mischievous goblin in German mythology, on account of the trouble it gave miners in refining it.

*This interview was conducted by Senior Editor Dominique Morneau*

